# UMAP Based Anomaly Detection for Minimal Residual Disease Quantification within Acute Myeloid Leukemia

**DOI:** 10.3390/cancers14040898

**Published:** 2022-02-11

**Authors:** Lisa Weijler, Florian Kowarsch, Matthias Wödlinger, Michael Reiter, Margarita Maurer-Granofszky, Angela Schumich, Michael N. Dworzak

**Affiliations:** 1 Computer Vision Lab, Faculty of Informatics, Technical University of Vienna, 1040 Vienna, Austria; lweijler@cvl.tuwien.ac.at (L.W.); florian.kowarsch@tuwien.ac.at (F.K.); mwoedlinger@cvl.tuwien.ac.at (M.W.); rei@cvl.tuwien.ac.at (M.R.); 2 Immunological Diagnostics, St. Anna Children’s Cancer Research Institute (CCRI), 1090 Vienna, Austria; margarita.maurer@ccri.at (M.M.-G.); angela.schumich@ccri.at (A.S.); 3 Labdia Labordiagnostik GmbH, 1090 Vienna, Austria

**Keywords:** acute myeloid leukemia, anomaly detection, UMAP, set-transformer, self-attention, flow cytometry, minimal residual disease, automated gating

## Abstract

**Simple Summary:**

Acute myeloid leukemia (AML) is the second most frequent leukemia entity in children and adolescents, and definitely the most aggressive variant. Multiparameter flow-cytometry is one of the methodologies most useful to monitor the number of remaining leukemic cells in bone marrow (minimal residual disease, MRD) in AML patients, because it is widely available and applicable to most patients. However, AML flow cytometry data show very complex patterns and identifying leukemic cells in the data is subjective, time-consuming and requires experienced operators who are not available world-wide. In this paper, we approach automatic assessment of AML flow cytometry samples with a novel semi-supervised machine learning model, leveraging implicit expert knowledge stored in a collection of manually assessed samples. Because AML data exhibit a high degree of variability in the patterns of blast cell populations that is difficult to model, the model detects anomalies starting from the appearance of normal cell populations.

**Abstract:**

Leukemia is the most frequent malignancy in children and adolescents, with acute lymphoblastic leukemia (ALL) and acute myeloid leukemia (AML) as the most common subtypes. Minimal residual disease (MRD) measured by flow cytometry (FCM) has proven to be a strong prognostic factor in ALL as well as in AML. Machine learning techniques have been emerging in the field of automated MRD quantification with the objective of superseding subjective and time-consuming manual analysis of FCM-MRD data. In contrast to ALL, where supervised multi-class classification methods have been successfully deployed for MRD detection, AML poses new challenges: AML is rarer (with fewer available training data) than ALL and much more heterogeneous in its immunophenotypic appearance, where one-class classification (anomaly detection) methods seem more suitable. In this work, a new semi-supervised approach based on the UMAP algorithm for MRD detection utilizing only labels of blast free FCM samples is presented. The method is tested on a newly gathered set of AML FCM samples and results are compared to state-of-the-art methods. We reach a median $F_{1}$-score of 0.794, while providing a transparent classification pipeline with explainable results that facilitates inter-disciplinary work between medical and technical experts. This work shows that despite several issues yet to overcome, the merits of automated MRD quantification can be fully exploited also in AML.

## 1. Introduction

Leukemia accounts for one-third of malignancy in children and adolescents, resulting in the most frequent childhood cancer (<18 years) [1]. The most common sub-types are acute lymphoblastic leukemia (ALL) and acute myeloid leukemia (AML), accounting for approximately 75% and 20%, respectively, [2]. Throughout the past decades, there has been a significant improvement of outcome for both ALL and AML, which is mostly attributable to improvements in diagnostic techniques and treatment protocols, superior risk-group stratified therapy, enhanced salvage at relapse, international collaboration as well as advances in supportive care. Despite the overall advancements, AML has a less favorable prognosis than ALL; the long-term survival rate for ALL has risen to approximately 90%, whereas for AML the survival rate is currently above 70% in Europe [1,3,4]. In addition, the relapse probability is higher for AML and chances of survival for relapsed patients remain at 30–40% [5,6,7]. A strong prognostic factor for treatment outcome and relapse risk is the minimal residual disease (MRD), which is defined as the fraction of remaining leukemic cells (blasts) at specific timepoints of treatment. MRD is an important measure to monitor treatment response, guide risk stratification and tailor treatment plans to individual disease patterns in order to provide the best possible outcome while minimizing toxicity of therapy [8,9].

A well established technique for MRD assessment is the detection of leukemia-associated immunophenotypes by multi-parameter flow cytometry (FCM-MRD) [10,11].

**Figure 1:**
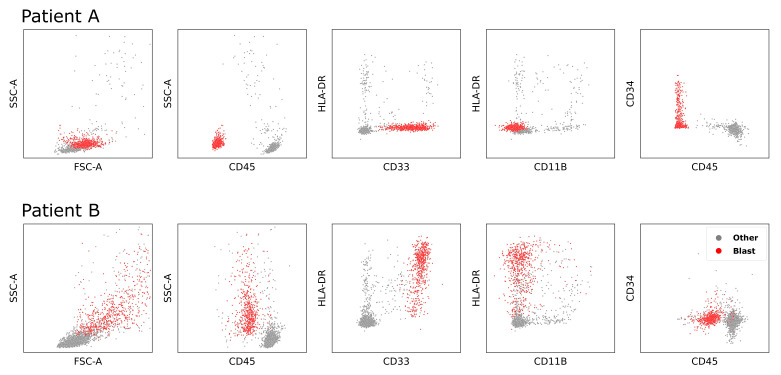
FCM-MRD data of two different patients. FCM data are typically visualized for manual gating by multiple 2D plots with different parameters as axis. Each dot represents the measurement vector of a single cell measured with a flow cytometer. Red dots denote leukemic cells and grey dots healthy cell populations. The Cluster of Differentiation (e.g., CD45) indicate antigens while SSC-A and FSC-A display the cell’s side and forward scatter. Note that the blast population varies in location and density between the different patients.

The amount of cells (events) per FCM-MRD sample varies from $10^{4}$ to $10^{6}$ and the proportion of blasts can be as low as 0.001%, turning MRD detection into searching for the needle in a haystack. Manual analysis of FCM-MRD data is based on gating, a process in which FCM experts select groups of events (i.e., cell populations) by drawing polygons around them in 2D scatter plots. For the assessment of one sample several different scatter plots are analysed, each showing a projection of the same sample onto different combinations of two features of the multi-dimensional data space. In order to identify the target populations multiple selections have to be combined by Boolean operations. Manual gating is resource-intensive, and subject to possible inconsistencies [12]. If cell populations exhibit a high degree of heterogeneity as in the case of AML, where the blasts vary in terms of shape, location and density between patients or even between different blast populations of the same patient (see Figure 1), manual gating becomes a very complex and error prone task.

In the background of the technological advancements of flow cytometers, capable of producing FCM data with more and more parameters resulting in high dimensional data spaces with complex distributions of events, numerous machine learning (ML) approaches have been developed to automate FCM data analysis, while manual gating is restricted to 3 features that can be visualized at once, automated gating can utilize the full multidimensional parameter space. Holistic approaches that take a whole sample as input are able to capture the spatial relation of cell populations to each other within the sample. These methods have been shown to be superior over classification methods learning fixed decision boundaries applicable to different samples [13,14]. A high degree of inter-sample variance in the shape and positions of sub-populations seems to be the reason for these results. An overview of state-of-the-art methods is given in Appendix A.

**Figure 2:**
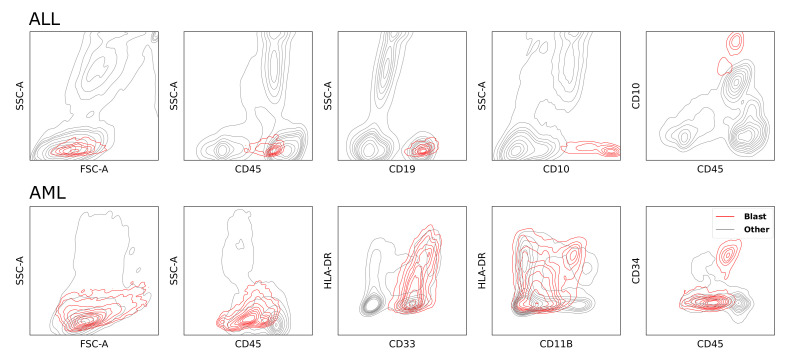
This figure shows the Gaussian kernel density estimation of events pooled together from randomly chosen FCM samples of ALL and AML patients. The density estimation of blasts is shown in red, for normal healthy cell populations grey is used. Blasts are a heterogeneous cell population with varying levels of heterogeneity for different acute leukemia sub-types. In AML samples the blast populations have a larger variance than in ALL.

However, with AML data there are additional challenges to overcome. AML is less common than ALL, which limits the use of supervised methods due to the scarcity of training data. Moreover, blast populations are much more heterogeneous than in ALL (see Figure 2). In addition to the standard staining panel (markers), sample or patient-specific drop-in markers are often required to correctly distinguish healthy from leukemic cells. Therefore the data spaces of AML samples are generally not restricted to the same set of features and the largest common set of features is not always sufficient to identify the blast population.

In this paper, a novel one-class classification approach based on Uniform Manifold Approximation and Projection (UMAP) [15] is presented for automated MRD assessment in FCM data of AML patients. It addresses the challenges mentioned above by omitting the training process and directly predicting leukemic blasts without the requirement of a pre-trained model. The core idea is to mix events of an FCM sample to be classified with randomly selected events of control samples, i.e., samples without blasts, and apply UMAP to separate cell populations into clusters. Clusters with little to no events of control samples are declared as blast clusters. Consequently, no labelled FCM data with leukemic cells are necessary, only labels of normal cell populations in control samples are used.

The Set-Transformer model [16], which is based on the self-attention mechanism, is suitable to model event distributions in higher dimensional data spaces. It can be considered as a supervised holistic approach and has been employed in our experiments as a successor model of [13,14]. We compare our proposed model with the Set-Transformer based model and outperform it by more than 300% (details of the Set-Transformer based classification on ALL data can be found in [17]).

UMAP has proven to find and preserve meaningful clusters in cell data while embedding the data in a lower dimensional space, where the multidimensional information is incorporated [18,19,20]. The low dimensional embedding allows for visual inspections throughout the prediction process leading to a transparent classification pipeline and explainable results that facilitate inter-disciplinary work between medical and technical experts.

## 2. Materials and Methods

In this work, a semi-supervised anomaly detection method for automated AML MRD quantification is presented using labels of healthy cell populations in blast-free samples only. Its main building blocks are UMAP and Hierarchical Density-Based Spatial Clustering of Applications with Noise (HDBSCAN) [21]. Our method is compared to the supervised approach based on self-attention blocks which is successfully applied to ALL data in [17]. The Set-Transformer, UMAP and HDBSCAN are introduced in Appendix B, where their suitability for our problem setting is discussed. An overview of the proposed classification pipeline and experimental setup is given in the following sections.

In Appendix B.6 we discuss the clustering of several well-established clustering methods within flow cytometry as an alternative to the clustering of UMAP and HDBSCAN.

### 2.1. UMAP-HDBSCAN Classification Pipeline

**Figure 3:**
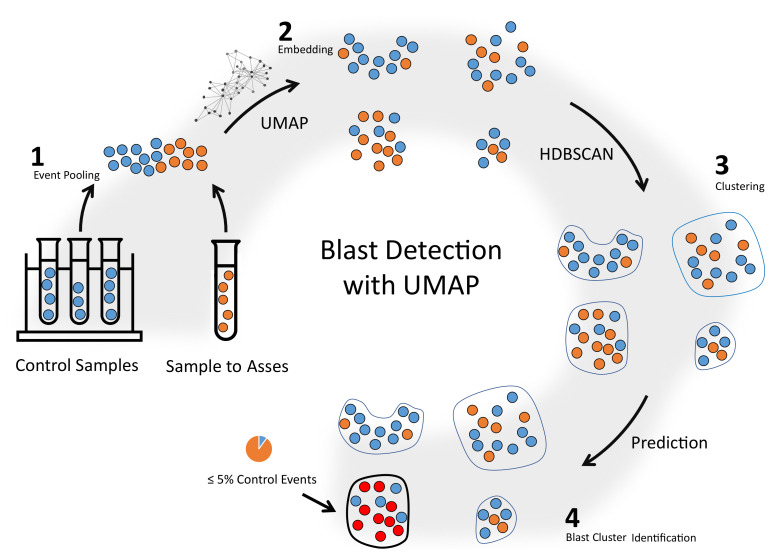
Schematic illustration of the method proposed for automated MRD detection in AML FCM data.

The method proposed is able to predict blasts in AML FCM data by using blast free samples (control samples) only. Blast detection is a binary classification task: each event of a sample is assigned either the label blast or non-blast. The presented classification pipeline is divided into four steps (as depicted in Figure 3):

1. A randomly selected subset of events coming from multiple MRD free control samples is mixed to the input FCM sample, which is to be assessed. The result is a mixed set of events.
2. The UMAP embedding of the mixed set is created. In this step, possible differences in the appearance of healthy cell populations in the control samples and the input sample are reduced by exploiting UMAP properties as explained in Appendix B.2.
3. Clusters are identified in the low-dimensional UMAP representation with HDBSCAN.
4. The clusters formed from cancer cells should not contain any or only a few events from control samples, as control samples do not contain blasts. Hence, clusters with a very low amount of control-events are most likely blast clusters. An empirical evaluation on hold-out samples led to a threshold of 5%. Test-events in those clusters are labelled as blasts, all others as non-blasts.

For a detailed description of the sampling strategy for control-events in the first step and reasoning for all parameters used see Appendix B.5.2.

### 2.2. Experimental Setup

In this section, the experimental setup is described. First, the data set and evaluation criteria are introduced and finally a brief overview of the experiments conducted is given.

#### 2.2.1. Data Set

Sampling and research was approved by local Ethics Committees, and informed consent was obtained from patients or patient’s parents or legal guardians according to the Declaration of Helsinki. Sample preparation and staining was done essentially according to international guidelines [9]. Per sample, a number of $5\times 10^{5}$ cellular events was acquired. For a detailed statistics on the number of cellular events per sample see Table A5.

The following data sets were used.

##### VIE MRD-Test Data

The data were collected between 2016 and 2021 at St. Anna Children’s Cancer Research Institute (CCRI) in Vienna using a Navios (Beckman Coulter, Brea CA, USA) flow cytometer. The samples were stained using customized dried format tubes (${\mathrm{DuraClone}}^{\mathrm{TM}}$, Beckman Coulter, Brea, CA, USA) and a dual tube approach. Both tubes contain eight fluorochrome-conjugated antibodies of which 5 are shared by both tubes (“backbone markers”: CD34, CD117, CD33, HLA-DR and CD45). The “leukemia associated immunophenotype- (“LAIP”)-tube” consisted of the following antibodies CD15 FITC/ CD34 ECD/ CD117 PC5.5/ CD33 PC7/ CD11b APC-Alexa750/ CD14 APC-Alexa700/ HLA-DR Pacific Blue/ CD45 Krome Orange plus patient specific drop-ins in PE and APC. Patient specific markers for optimal discrimination of leukemic blasts from normal regenerating cells are determined at the time of diagnosis and used in the follow-up for MRD detection and quantification [11]. The “colony formation unit (“CFU”)-tube” consisted of the following antibodies: CD38 FITC/ CD34 ECD/ CD117 PC5.5/ CD33 PC7/ CD45RA APC-Alexa750/ CD123-APC Alexa700/ HLA-DR Pacific Blue/ CD45 Krome Orange. For drop in markers in the CFU tube, CD371 PE and CD99 APC were used. For full details on antibodies see Table A1. We used a total of 66 data files (LAIP, *n* = 37; CFU, *n* = 29) from 10 patients with positive MRD levels from different timepoints of therapy. For a full listing of timepoints of therapy present in the data set see Table A2. Table A3 and Table A4 give details on clinical and biological patient characteristics as well as the LAIPs identified per patient.

##### VIE Control-Control Data

To obtain MRD negative control samples, we stained bone marrow (BM) samples of pediatric patients without any history of myeloid malignancy. In addition we used BM samples from pediatric patients with AML at a later stage of therapy and with proven MRD negativity (via flow cytometry and/or molecular methodology (RT-PCR)). The data were collected between 2015 and 2021 at St. Anna Children’s Cancer Research Institute. Samples were stained as indicated above using LAIP and CFU tubes. We used a total of 80 data files (LAIP, *n* = 43; CFU, *n* = 37).

The events of all samples in the two data sets were labelled using manual gating by at least two experts to obtain objective and reliable ground-truth data. Whenever available, results were confirmed using an independent molecular methodology (RT-PCR). Kaluza 2.0 software (Beckman Coulter, Brea, CA, USA) was used for manual data analysis. All obtained samples are manually gated following the same procedure. First the events are filtered by excluding non-viable cells, debris and doublets. Remaining events (cells) are selected based on expression of CD45 in a gate called *Denominator* and contain both, normal healthy cells as well as blasts (if present in the sample). Based on expression of the stem cell marker CD34, blasts can be divided in CD34 positive or CD34 negative blasts. The latter are further defined as events with low side scatter (SSC) properties and as such fall in the so called *bermude area* on the CD45/SSC bi-dimensional plot. Cells within the bermude area are further categorized in monocytes, granulocytes, proerythrocytes or promyeloctyes, after exlusion of basophiles, plasmacytic dendritic cells, mast cells and plasma cells.

The resulting output of a sample analysed by FCM is a matrix $E\in R^{N\times m}$, where each row corresponds to a single cell (event). The number of rows *N* denotes the number of measured events, which can be different for every sample, and the number of columns *m* denotes the number of features extracted. Each sample is compensated and transformed with a logical transformation in a pre-processing step.

For LAIP tubes drop-in markers are sample- (patient-) specific and mostly not present in control samples. Those have to be omitted, as the input vectors need to be of same length and corresponding to the same features for the experiments in this paper. On the contrary, for CFU-tubes drop-ins are fixed and can therefore be included.

#### 2.2.2. Evaluation

For the evaluation of experiments precision *p*, recall *r* and $F_{1}$-score $F_{1}$ are calculated on a single event basis for every FCM sample in the test set:

$$p=\frac{TP}{TP+FP},\;r=\frac{TP}{TP+FN},\;F_{1}=\frac{2TP}{2TP+FP+FN},$$

with TP as number of true positive events (blasts identified as blasts), FP as false positive, and FN as false negative events in the classification of a FCM sample. The final scores used for comparison between experiments are determined by taking the mean and median of precision *p*, recall *r* and $F_{1}$-score $F_{1}$ over the samples of the test set. If the number of blast cells $n_{blasts}$ in a sample is below a threshold, the sample is considered as MRD negative. According to international standards this threshold is 50 in the case of AML-MRD. If the method predicts $n_{blasts}<50$ cells in a MRD negative sample precision, recall and $F_{1}$-score are set to 1.

#### 2.2.3. Experiments

Four different experiments for MRD quantification in FCM data of AML patients are conducted. First, we apply the supervised method based on the self-attention mechanism of the Set-Transformer. Then the method proposed is tested and the impact of pre-filtering of events as well as variations of control samples is explored. A detailed description of all experiments conducted is given in Appendix B.5.

## 3. Results

In this section, the results of the experiments are presented. Average precision (p), average recall (r), average F-Score (avg $F_{1}$), and median F-Score (med $F_{1}$) are used for comparison as explained in Section 2.2.2. Results for experiments, where tube-specific marker were used, are given seperatly for CFU and LAIP as well as combined (CFU and LAIP).

### 3.1. Set-Transformer

The approach based on the self-attention mechanism (see [16]) works well for MRD detection in ALL [17]. For AML, however, results are rather disappointing (Table 1). Restricting the marker panel to the 5-color backbone (BB) panel in order to increase training data yields worse results than exploiting the 8-color CFU (+ drop-ins) and LAIP tube panel. When looking at the results for the tubes separately no big difference is noticeable. The low scores could be attributed to a data problem as very little training and validation data are available (see Table A6), though in [17] it is shown that for ALL the approach works for training and validation sets as small as 10 samples. This highlights the additional challenges present in AML MRD quantification, namely a more limited data availability in combination with a more heterogeneous target population. Besides, while for the experiments in [17] data from the same timepoint (day 15 after induction therapy) were taken, the scarcity of AML FCM data demands to include FCM samples from different stages during and after therapy leading to additional varieties of healthy cell populations.

### 3.2. UMAP-HDBSCAN Classification Pipeline

**Figure 4:**
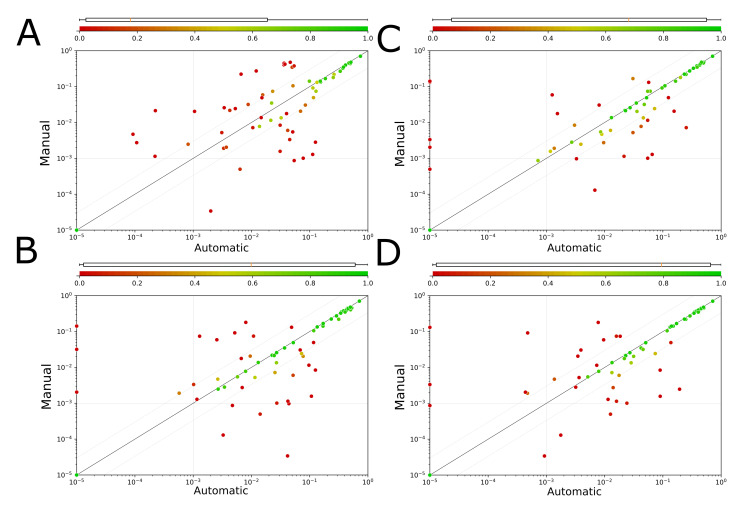
$F_{1}$-scores and predicted MRD values of automatic assessment compared to the manually obtained ground truth. Each dot represents a single sample, the position encodes the MRD values and the color the $F_{1}$-score of a sample. Predictions that are within the range of either less than 3 times or more than 1/3 of the true MRD are considered as acceptable (correct) predictions [22]. These so called concordance margin is visualized as gray lines around the first median. The plots are partitioned into four quadrants by the threshold of 0.1% (thin vertical and horizontal lines), which is the lower clinically relevant resolution. Each plot shows the results of a different experiment using the 8-color tube-specific panels plus drop-ins for CFU samples (**A**): Set-Transformer (Table 1), (**B**): UMAP-HDBSCAN Classification Pipeline (Table 2), (**C**): UMAP-HDBSCAN Classification Pipeline with Pre-Filtering (Table 3), (**D**): UMAP-HDBSCAN Classification Pipeline—Variation of Control Samples (Table 4). Samples that are considered as negative (less than 50 Blast events) are located at the origin of coordinates and occur in following quantity: (**A**): 3 (**B**): 1 (**C**): 2 (**D**): 2. The samples where the automatic assessment failed to detect any MRD despite an MRD of >0.01% are samples of the LAIP-panel and thus lack of patient-specific drop-in markers. Those drop-in markers are important for those samples to separate blasts from non-blasts. Consequently, the blasts are not separated from normal, healthy cells in the UMAP embedding and do not get assigned their own cluster by HDBSCAN.

With only 15 control samples, the proposed classification pipeline, reaches a 1.44 times better performance than the Set-Transformer approach with respect to average $F_{1}$-score; the median $F_{1}$-score triples. When looking at the results for CFU and LAIP tubes separately, it is revealed that LAIP tube samples perform worse (Table 2). Figure 4 shows the sample-wise results of the proposed classification pipeline in B compared to the Set-Transformer in A.

**Figure 5:**
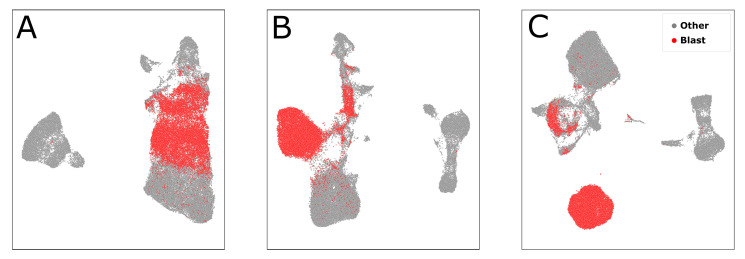
UMAP embedding of a MRD positive FCM sample using (**A**) 5-color backbone panel, (**B**) the 8-color LAIP panel, and (**C**) the full panel (LAIP plus drop-in markers). UMAP can only properly separate blasts in the embedding space given the drop-in markers. We cross-checked several UMAP embeddings with medical experts in cases where UMAP has a very clear separation of blast clusters but some remaining blasts mixed together with healthy cells. The blast gates of manual gating often were drawn through very dense areas, where slight shifts can have a big effect in terms of absolute numbers of blasts. In most cases (such as in (**C**)), subsequently correcting the gates also resulted in more coherent clusters consistent with UMAP.

Due to the transparency of our method samples can be inspected at any stage of the pipeline. We take advantage of this property and examine samples that score $F_{1}\leq 0.5$. The main issue identified is the lack of drop-in markers. In some cases blasts cannot be sufficiently separated from non-blasts in the embedding space without taking into account those additional markers. Figure 5 shows the UMAP embedding of test samples using backbone markers only, tube-specific markers and tube-specific + drop-in markers. Based on the examination of those plots, approximately 25% of all LAIP samples require the additional drop-in markers to separate blasts from non-blasts in the UMAP embedding space. For CFU samples the drop-ins are fixed and can thus be included as explained in Section 2.2.1. This explains the performance differences between LAIP and CFU tube samples.

**Figure 6:**
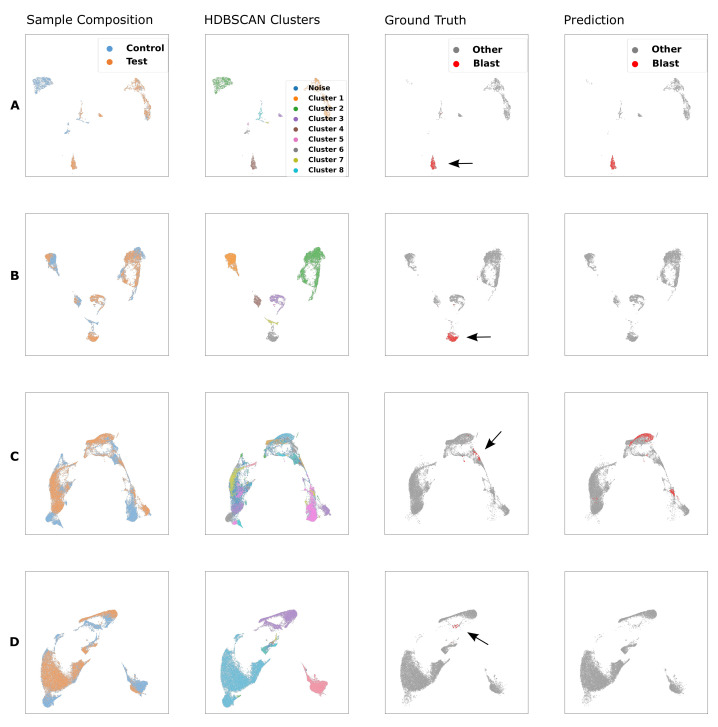
Each row corresponds to one FCM sample that is analysed. The first column shows the composition of test- and control-events, the second the clusters detected by HDBSCAN (each color defines one cluster), the third column shows the embedding of the test sample only and the blasts found by manual gating (arrowheads point at blasts) and finally, the last column the detected blasts by the method proposed, while sample (**A**) is a prime example, where blasts form a separate cluster that is correctly detected, (**B**–**D**) are examples for the issues identified. (**B**) shows a sample, where blasts form a separated cluster, yet it was not detected as the percentage of control-events in this cluster was a bit above the 5% threshold. (**C**) is an example for false positive clusters. The blast cluster was correctly identified but others as well due to poor coverage of healthy cells and a finer HDBSCAN clustering. Finally, (**D**) is a sample where blasts are not well separated in the embedding space due to missing drop-in markers and hence blasts are not detected as single cluster.

Other issues identified are false positive clusters when healthy cell populations are not sufficiently covered by control-events. Since drop-in markers are included for CFU samples, for most samples blasts form a seperated cluster in the UMAP embedding space and are correctly identified as blasts. However, additional false positive clusters arise, which is also noticeable in the low precision p=0.572 but high recall r=0.812. False positive clusters can arise in combination with microclusters, where HDBSCAN predicts several smaller clusters of one cell population. Microclusters are no problem in general as long as control-events are well distributed. However the smaller the clusters, the more likely it is to miss out on control-events and hence to have false positive clusters. Additionally, we found that for samples where blasts form a clear, separated cluster in the embedding space, the threshold of 5% control-events used to predict blast clusters can be a source for false negatives. An example of each issue identified as well as an example for perfect classification is given in Figure 6.

### 3.3. UMAP-HDBSCAN Classification Pipeline with Pre-Filtering

As stated in Section 2.2.1 the bermude area is not a single cell population but is comprised of various different cell populations defined by small side scatter properties and rather dim to medium CD45 expression. This implies that the bermude area is much more heterogeneous than a single cell population such as the CD34 positive cells. This is reflected in the $F_{1}$-scores of the pre-filtering; predicting CD34 positive cells with the Set-Transformer architecture yields higher results than predicting events in the bermude area. When comparing the outcome obtained with tube-specific markers and backbone markers, it is revealed that for this classification backbone marker are sufficient. In the case of predicting events in the bermude area, more training data provide better results than more markers (Table 5).

When applying the proposed method to the distilled test sample overall mean and median $F_{1}$-score improve in comparison to the unfiltered experiments. Looking at the tube results separately, we can see an increase for LAIP samples, whereas for CFU samples a slight decrease is noticeable (Table 3). The performance increase for LAIP samples is attributable to the fact that pre-filtering reduces variations of healthy cells and hence counteracts false positive clusters. The remaining samples for which MRD prediction fails are mainly those, where blast are not sufficiently separated from non-blasts in the embedding space due to missing drop-in markers. With respect to CFU samples, precision has improved as false positive clusters have decreased. The slight performance drop is explained by the additional layer of potential error added by the pre-filtering process. On average the prediction of CD34 positve events and those in the bermude area is worse for CFU than LAIP samples and the additional layer of error has thus more effect on CFU samples. Figure 4C shows the sample-wise results of the proposed classification pipeline with pre-filtered events.

### 3.4. UMAP-HDBSCAN Classification Pipeline-Variation of Control Samples

One way to cope with variations of healthy cell populations is to reduce them beforehand in a pre-processing step like in the previous experiment. Another way would be to improve the coverage of possible variations by using more control samples. We test our method for double as many (30) and all control samples available. The performance for LAIP samples increases using 30 control samples instead of 15 but stagnates when adding more. For CFU samples we see a slight drop when increasing the number of control samples. When examining the results for each element in the test set separately, it is revealed that the samples that yield the lowest scores are the same as with 15 control samples with minor fluctuation. Looking at the results for both tubes combined adding more control samples shows a similar result as the pre-filtering experiment above, with a lower mean $F_{1}$-score but higher median $F_{1}$-score.

**Figure A3:**
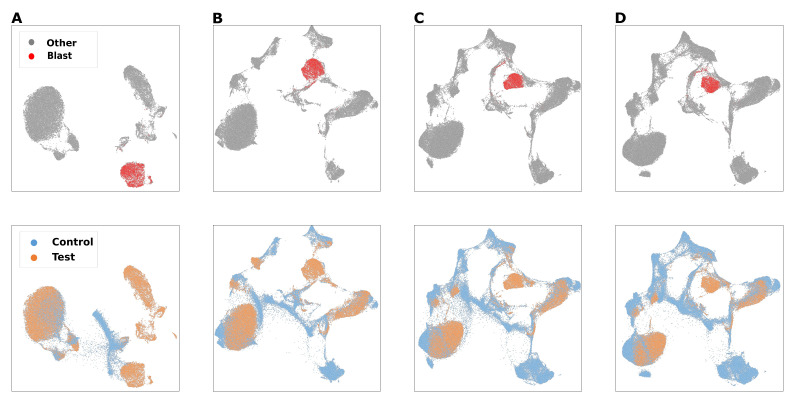
UMAP embedding of a test sample mixed with control-events for a fixed transform_ratio=1 but varying control_ratio∈{0,0.5,1,1.5} (columns **A**–**D**). The first row shows the embedding learned with increasing number of control-events mixed to the test sample. Blasts are in red, non-blast events are grey. The second row shows the same respective UMAP representation with the test-events (orange) where additional control-events (blue) are transformed into the embedding space. For low value of control_ratio blast events are well separated but additionally projected control-events are spread out between clusters (column **A**–**B**). For higher values of control_ratio blasts adhere to healthy cell populations, which makes it harder to detect them as separate cluster, but additionally projected control-events mix well with the clusters of healthy cell populations.

With respect to the question whether capturing more variety within one sample or between samples is of bigger importance, the results indicate that it is more beneficial to include less events from more control samples than vice versa. Nevertheless, we observed that with increasing number of control samples the control-events additionally transformed into the embedding space tend to be more spread between existing clusters, which is explicable by the following. The number of events selected per sample decreases when more control samples are added. If a cell population is rather heterogeneous within one sample, the additionally selected events run risk to be too different and to not adhere to the healthy cell populations already embedded similar to as demonstrated in Figure A3. This can lead to false positive clusters as well as false negative clusters if those “lost” control-events contaminate blast clusters and hence can have a slight reverse effect than intended.

Figure 4 shows the MRD detected with the methods above versus MRD determined by manual gating for each test sample (CFU and LAIP).

## 4. Discussion

In this work, a novel method based on UMAP and HDBSCAN for MRD detection in AML FCM samples is introduced. The approach does not require model-training and only uses control samples that do not contain leukemic cells. It falls in the category of one-class classification approaches, since only labels of healthy cell populations in control samples are utilized. We compare our method to a supervised approach based on the Set-Transformer that has proven to be successful for ALL data [17]. With only 15 control samples our approach reaches an average $F_{1}$-score of 0.514 and median $F_{1}$-score of 0.595. Those results outperform the Set-Transformer approach by more than 40% and 200%, respectively. The low scores of the Set-Transformer (avg $F_{1}=0.356$, med $F_{1}=0.177$) are an indication that one-class classification methods are more suitable than supervised methods for MRD detection in AML due to data scarcity and heterogeneity of blast populations. By removing events from the test sample in a pre-processing step, the search for blasts can be narrowed down, which further improves performance reaching an average $F_{1}=0.557$ and median $F_{1}=0.680$. The main issue identified was that sample-specific drop-in markers are omitted, which can be crucial for separation of blast and non-blasts in the UMAP embedding. When exploring the impact of additional control samples it was found that selecting events from a bigger pool of control samples can counteract the prediction of false positive cluster by covering more inter-sample variations of healthy cell populations but should be used with caution as it can lead to an opposite effect due to the sampling strategy. Overall, it yields performance improvements similar to reducing possible variations by pre-filtering. A core functionality of UMAP is visualizing high-dimensional data, though in this work it is a crucial part of the classification pipeline proposed. The visualization, however, is a pleasant side aspect that makes the whole classification process transparent and interpretable. The resulting classification of events is comprehensible and facilitate inter-disciplinary work between medical and technical experts.

### 4.1. Limitations

The method presented is subject to some limitations that we outline in this section.

Since the sampling of control-events is a random procedure and UMAP as well as HDBSCAN are non-deterministic algorithms, results can differ slightly from run to run. The variability of results can be reduced by setting a seed-value or repeating experiments several times and looking at the average of results.

As discussed in Section 3.2 and depicted in Figure 5 drop-in markers are necessary in some samples to facilitate blast separation in an UMAP embedding. It is assumed that the drop-in markers entail additional information for blast separation in high dimension and are therefore useful beyond their application in 2D gating scatter plots. Drop-in markers are utilized in the manual gating process, not using them in an automated process neglects helpful information and is therefore a major limitation of the proposed approach.

### 4.2. Future Work

Given that UMAP is a graph-based algorithm and given its potential to form biological meaningful clusters, Graph Neural Networks (GNN) seem worthwhile exploring. The graph constructed by UMAP, which is an approximation of and thus inherits information of the manifold the data relies on, could be used as input of a node classification or anomalous subgraph detection task. This would be an holistic approach since not only the feature vectors but also the relation of cell populations within one sample are taken into account through the node connectivity matrix of the graph.

As mentioned in Appendix B.5.2 and Section 3.4, the proposed method relies on effective sampling from a data base of control-events. The main goal is to sample events of the control cases, such that the resulting event pool is as representative as possible describing all possible data variations. This objective is tried to be fulfilled by sampling a fixed relative amount of events per labeled cell population from each sample. However, such sampling strategies still over present the dense regions of each cell population and are therefore not optimal regarding data variation. Sampling with respect to maximum mean discrepancy (MMD) as described in [23] could be one way to enhance the sampling process. Selecting events from a sample while minimizing the squared MMD yields selected events whose distribution approximates the sample’s data distribution.

Currently, the fixed global threshold of the proportion of control-events to detect blast clusters represents a limitation of the method. It could be beneficial to use an adaptive sample specific threshold determined by the statistics of the control-events proportion of all clusters of a sample.

Another promising branch of future development is to utilize all drop-in markers of a sample during processing. Utilizing all drop-in markers requires to take the relation between events without drop-in markers (e.g., from control samples) to neighbouring events with drop-in markers (e.g., from test samples) into account. A common way is to predict an event’s unknown marker expression level by nearest neighbour imputation based on the overlapping markers [24,25,26,27,28]. Such approaches assume that each event is nearly identical to the nearest neighbouring events. Possible UMAP based solutions could either merge two separately learnt embeddings (one using control- and test-events with tube-specific markers, the other only test-events with all markers) or optimize one embedding using both data compositions simultaneously. The later could be accomplished by the semi-supervised capabilities of parametric UMAP [29].

## 5. Conclusions

We propose a method that only avails itself of healthy cell populations, is easy to implement and provides transparency and interpretability through out the classification process.

By exploring alternative clustering methods commonly used within flow cytometry we found that the push-pull characteristic of UMAP together with its use of local distance metric is crucial for the success of our pipeline.

Further, the low scores of the Set-Transformer are an indication that one-class classification methods are more suitable than supervised methods for MRD detection in AML due to data scarcity and heterogeneity of blast populations.

When looking at the results in combination with run-time we recommend pre-filtering of the events prior to classification as presented in Section 3.3. While including more control samples yields similar results, pre-filtering also brings the benefit of superior run-time.

We demonstrate that despite the challenging nature of MRD detection in AML, automated gating is not just a future dream but realistically feasible.

On a general note, we want to emphasize the importance of international and inter-laboratory collaboration in combination with the establishment of standards for data acquisition, marker panels, gating strategies and flow cytometry settings, in order to produce comparable FCM data across laboratories and countries. We believe that future steps in this direction are crucial for increasing data availability and hence for the development and maintenance of high quality automatic MRD detection methods.

## Figures and Tables

**Table 1:** Experimental results for the supervised approach based on the Set-Transformer (ST) [17]. A patient cross validation was performed and thus the train and validation set change per patient. Experiments were conducted for the backbone marker (BB) as well as the full 8-color LAIP and CFU panel.

Experiment	N-Test	*p*	r	Avg $F_{1}$	Med $F_{1}$
ST-LAIP	37	0.392	0.468	0.356	0.155
ST-CFU	29	0.404	0.448	0.357	0.186
ST-CFU and LAIP	66	0.398	0.459	0.356	0.177
ST-BB	66	0.335	0.465	0.330	0.107

**Table 2:** Results of the proposed classification pipeline. For CFU and LAIP tubes separately and combined.

Experiment	N-Test	N-Control	*p*	r	Avg $F_{1}$	Med $F_{1}$
UMAP-LAIP	37	15	0.563	0.462	0.443	0.253
UMAP-CFU	29	15	0.572	0.812	0.607	0.880
UMAP-CFU and LAIP	66	15/15	0.567	0.612	0.514	0.595

**Table 3:** Results of the proposed classification pipeline with pre-filtered events for CFU and LAIP tubes separately as well as combined.

Experiment	N-Test	N-Control	*p*	r	Avg $F_{1}$	Med $F_{1}$
UMAP-LAIP	37	15	0.624	0.562	0.526	0.680
UMAP-CFU	29	15	0.630	0.763	0.599	0.730
UMAP-CFU and LAIP	66	15/15	0.627	0.649	0.557	0.680

**Table 4:** Results of the proposed classification pipeline for varying number of control samples.

Experiment	N-Test	N-Control	*p*	r	Avg $F_{1}$	Med $F_{1}$
UMAP-LAIP	37	15	0.563	0.462	0.443	0.253
UMAP-LAIP	37	30	0.570	0.538	0.514	0.793
UMAP-LAIP	37	43	0.593	0.525	0.505	0.794
UMAP-CFU	29	15	0.572	0.812	0.607	0.880
UMAP-CFU	29	30	0.566	0.765	0.596	0.785
UMAP-CFU	29	37	0.552	0.774	0.587	0.805
UMAP-CFU and LAIP	66	15/15	0.567	0.612	0.514	0.595
UMAP-CFU and LAIP	66	30/30	0.568	0.636	0.549	0.793
UMAP-CFU and LAIP	66	37/43	0.575	0.632	0.540	0.794

**Table 5:** Results of predicting CD34 positive events and those in the bermude area (CD34 neg) with the Set-Transformer (ST). For tube-specific results, using the 8-color LAIP and CFU (+drop-ins) panels the number of test and training samples are given separately. 20% of the training data are used for evaluation.

Experiment		N-Test	N-Train	*p*	r	Avg $F_{1}$	Med $F_{1}$
ST-CD34 pos	BB	66	80	0.890	0.918	0.889	0.914
	tube-specific	29/37	37/43	0.927	0.874	0.884	0.914
ST-bermude	BB	66	80	0.687	0.890	0.742	0.779
	tube-specific	29/37	37/43	0.632	0.896	0.702	0.720

**Figure A1:**
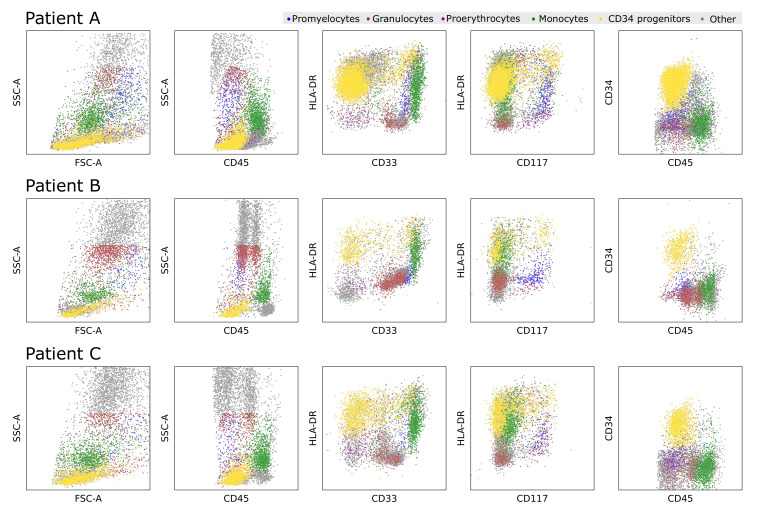
Healthy cell populations exhibit low variation between patients. Each row shows an FCM sample of a blast-free patient taken at the same stage during therapy (after the third cycle of consolidation therapy). Differences in density as well as minor shifts of locations are noticeable. Colors of events falling into the bermude area represent promyelocytes (blue), granulocytes (brown), proerythrocytes (purple), monocytes (green), CD34 progenitors (yellow).

**Figure A2:**
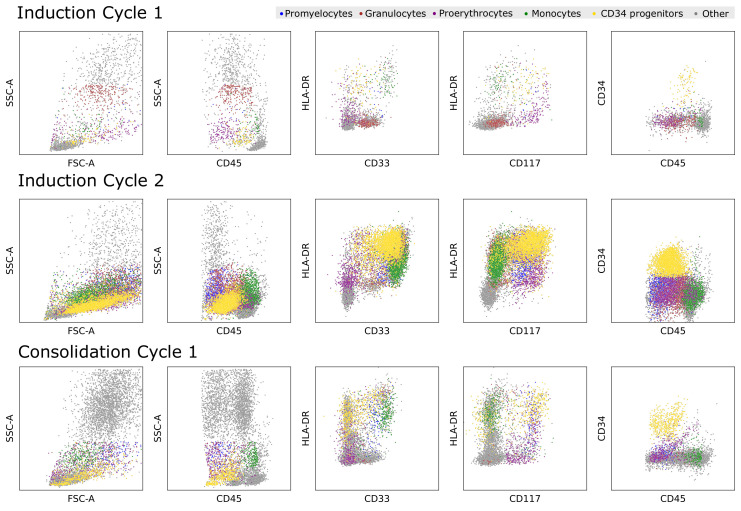
FCM samples of one patient from different stages during therapy are compared. Therapy mainly affects the density of different cell populations. Colors of events falling into the bermude area represent promyelocytes (blue), granulocytes (brown), proerythrocytes (purple), monocytes (green), CD34 progenitors (yellow).

## Data Availability

The used FCM data samples can be requested from the authors.

## Appendix A. Related Work

Several approaches have been developed for automated FCM data analysis to replicate or aid manual data analysis. A distinction can be made between discovery and targeted analysis; while the first aims at discovering novel unknown cell populations, the latter aims at detecting well-defined, known ones. Since MRD detection falls into the category of targeted analysis, we focus on giving an overview of automated targeted FCM data analysis. Additionally a brief summary of dimensionality reduction methods is given, since those are not only used to aid visualization but also for feature extraction as pre-processing step before targeted analysis. For a more comprehensive review of current trends in automated FCM data analyis the reader is referred to [30].

### Appendix A.1. FCM Analysis with Statistical Methods

In [31], FCM samples are represented as probability density functions which are then matched to a set of reference samples. Other methods parametrize populations in FCM samples with mixture models [13,14,32,33,34,35]. These methods model cell populations in samples with mixture models and then compare the resulting distribution with a set of reference samples where gating information is available. In [14], Gaussian Mixtures are fitted to a sample population with expectation maximization. An improved version with a closed form optimization in the fitting process is proposed in [13]. In SWIFT [33] Gaussian Mixture Models are combined with a weighting sampling procedure to improve discrimination of rare sub-populations. BayesFlow [35] introduces a hierarchical Bayesian model which allows expert knowledge to be incorporated through informative priors. Diffcyt [36] uses unsupervised clustering in combination with supervised statistical analyses, namely empirical Bayes moderated tests adapted from transcriptomics for differential analysis, to detect cell populations. Another state-of-the-art approach is Citrus [37], which uses hierarchical clustering and regularized regression to predict the endpoint of interest for each sample.

### Appendix A.2. FCM Analysis with Neural Networks

Recently, methods that employ neural networks have been introduced to identify populations in FCM data. In [38,39] recent developments in Convolutional Neural Networks (CNNs) are applied to imaging FCM applications. For non-imaging FCM data initial works processed the samples cell-wise [40,41,42]. CellCNN [43] uses a 1D-convolution layer to project the measurements of each cell to an embedding space then applies a pooling layer to aggregate information in order to learn the associated phenotype from multi-cell input. In [40,41], the problem is cast as a binary classification problem. In [42], Li et al. check the similarity of a given sample with a set of reference samples and then train a four layer network on the best match. However, these methods can only learn fixed decision boundaries to separate biologically meaningful sub-populations and can not adapt to variations between samples. A way to circumvent this and process whole samples instead of single cells is proposed in [44]. Here self-organizing maps are employed to obtain a 2D image from a given FCM sample. These images are then processed using CNNs, trained in a supervised manner. Another method that allows processing full samples is presented in [17] where a neural network based on the transformer architecture [45] is proposed, that allows processing of a full sample in a single neural network forward pass.

### Appendix A.3. Dimensionality Reduction for FCM Analysis

Initial work for general dimensionality reduction is typically based on linear transformations applied to the data to reduce correlations between features directions. In principle component analysis (PCA) [46] features are projected onto a lower dimensional linear subspace in which only high variance directions are retained. Besides PCA, many other dimensionality reduction techniques have been proposed that allow nonlinear transformations. In t-SNE [47] high dimensional data are transformed into a lower dimensional space by constructing a probability distribution over pairs and assigning a high probabilty to similar and a low probability to dissimilar points. t-SNE has been shown to be capable of stratifying general cellular lineages in mass cytometry data [48]. More recently, the uniform manifold approximation and projection for dimension reduction (UMAP) algorithm has been proposed [15]. UMAP creates qualitatively comparable dimensionality reduction when compared to t-SNE with significantly reduced run times in most cases [15]. It has been shown to work with single-cell data [18] and has been applied successfully to FCM data in [20].

## Appendix B. Methods

In this section, we introduce the Set-Transformer, UMAP and HDBSCAN; we discuss why they are a reasonable fit for our problem setting. Additional information of the dataset used is given and a detailed description of the experimental setup is provided. Finally, technical and computational details are presented.

### Appendix B.1. Set Transformer-Attention

The original transformer architecture was proposed in 2017 [45]. It allows to learn from sequences (of variable length) without recurrence. The key novelty in this architecture is the introduction of self-attention which allows capturing global contextual information between elements in a sequence. However, the attention mechanism entails a quadratic complexity in the input length $O(n^{2})$ of both memory and time. Albeit processing a whole sample at once and capturing global information are favorable properties, this is hardly applicable for FCM data analysis, where samples contain up to $10^{6}$ events (=sequence length).

Fortunately, several models have emerged that aim to reduce complexity. The most related to the application of FCM data analysis are Set-Transformers proposed by Lee et al. [16] that explicitly treat the input as an order-invariant set. This resonates with our problem setting since the order of events in a sample is not relevant. Instead of standard multi-head self-attention, Set-Transformers introduce the Induced Set Attention Block (ISAB) based on the idea of inducing points from the theory of Gaussian processes. With this modification the complexity becomes linear in the sequence length O(N). For a more detailed description of how the Set-Transformer can be applied to FCM data the reader is referred to [17].

### Appendix B.2. UMAP

Initially proposed by McInnes et al. [15], UMAP consists of two steps:

1. Under the use of a custom local distance metric a neighbourhood graph is constructed to form a topological representation of the high dimensional data.
2. A low dimensional embedding of the data is obtained such that the representing graph of the data points in the embedding is as structurally similar as possible to the graph in high dimension.

The initial representation of the high dimensional data is formed by a weighted graph in which the edge weights represents the likelihood that two data points are connected. The process of graph construction makes use of tools from Riemannian geometry, which allows to take computational efficient shortcuts at runtime and justifies chosen characteristics of the algorithm. The constructed graph is then projected into the low dimensional space. In contrast to the high dimensional space, where the edge weight are defined by the custom local distance metric, in the embedding space the edge weights reflect the euclidean distance between data points. The low dimensional projection is optimized via a force-directed graph layout algorithm.

The varying notion of distance that was created to approximate the manifold on which the data lies is translated into euclidean distance. This translation reveals the inherent structure of the data in euclidean distance with respect to the global coordinate system. In our setting, this behaviour of the algorithm is exploited to smooth out possible inter-sample variances. The local connectivity of the manifold introduced by the custom local metric makes sure no point is completely isolated, i.e., each point is connected to at least one other point. This property in combination with the force-directed graph layout algorithm pulls similar cell populations of different samples closer together in the embedding space. This consequently improves clustering performance compared to directly cluster unprocessed high dimensional data as empirically demonstrated on image data in [49].

### Appendix B.3. HDBSCAN

HDBSCAN [21] is an adaption of DBSCAN [50], which is a density based clustering algorithm. Clusters are formed among data points in dense regions, while data points in sparse areas are classified as noise. DBSCAN uses a density based distance metric to build a single linkage clustering dendrogram. In order to obtain cluster assignments the dendrogram is cut according to a distance parameter ϵ. HDBSCAN eliminates ϵ by introducing min_cluster_size, which defines the minimum number of data points for a single linkage split to be considered as a cluster. This allows to asses the stability of clusters in the dendrogram, which can be used to cut the tree at varying heights resulting in clusters with possible varying density. In addition min_cluster_size is designed to be more intuitively pickable for the user.

We use HDBSCAN to identify clusters in the UMAP projection. The following aspects indicate that HDBSCAN is a suitable clustering algorithm for the problem stated. Since clusters are not necessarily spherical, density based clustering is a better fit than algorithms like *k*-means [50]. Ideally, the clusters represent biological meaningful cell populations and hence we want to be able to identify clusters of varying density. Further, HDBSCAN does not require to specify the number of clusters in advance, which benefits its use in an automated pipeline. In addition, HDBSCAN classifies data points in sparse areas as noise, which can partly compensate artefacts produced by the UMAP.

### Appendix B.4. Data Set

Table A1 and Table A2 give further details on the data set used with respect to antibodies of the marker panels and timepoints of therapy, respectively. Detailed patient characteristics and LAIPs identified are given in Table A3 and Table A4. Table A5 gives an overview of the amount of nucleated events as well as the MRD percentage per sample in the data set used.

**Table A1:** Full details on antibodies used.

	Antigen	Fluorochrome	Clone	Source
LAIP	CD15	FITC	80H5	Beckman Coulter
(${\mathrm{DuraClone}}^{\mathrm{TM}}$)	CD34	ECD	581	Beckman Coulter
	CD117	PC5.5	104D2D1	Beckman Coulter
	CD33	PC7	D3HL60.251	Beckman Coulter
	CD14	APC-Alexa700	RMO52	Beckman Coulter
	CD11b	APC-Alexa750	Bear1	Beckman Coulter
	HLA-DR	Pacific Blue	IMMU-357.12	Beckman Coulter
	CD45	Krome Orange	J33	Beckman Coulter
CFU	CD38	FITC	T16	Beckman Coulter
(${\mathrm{DuraClone}}^{\mathrm{TM}}$)	CD34	ECD	581	Beckman Coulter
	CD117	PC5.5	104D2D1	Beckman Coulter
	CD33	PC7	D3HL60.251	Beckman Coulter
	CD123	APC-Alexa700	SSDCLY107D2	Beckman Coulter
	CD45RA	APC-Alexa750	2H4LDH11LDB9	Beckman Coulter
	HLA-DR	Pacific Blue	IMMU-357.12	Beckman Coulter
	CD45	Krome Orange	J33	Beckman Coulter
Drop-in	CD7	PE	MEM-186	Exbio
markers	CD11a	PE	MEM25	Exbio
	CD19	PE	LT19	Exbio
	CD56	PE	LT56	Exbio
	CD371	PE	50C1	BioLegend
	NG2	PE	7.1	Beckman Coulter
	CD13	APC	WM15	Exbio
	CD71	APC	MEM-75	Exbio
	CD99	APC	3B2/TA8	Exbio

**Table A2:** Details on different timepoints of therapy that are present in the data set used.

Timepoints VIE Control	Abbreviation	Therapy Protocol	No. of Samples
after 1st Induction	IND1/IND12	BFM-AML ^1^	6
after 2nd Induction	IND2	BFM-AML ^1^	12
after Consolidation 1	CON1	BFM-AML ^1^	14
after Consolidation 2	CON2	BFM-AML ^1^	4
after Consolidation 3	CON3	BFM-AML ^1^	2
End of therapy	End	BFM-AML ^1^	4
Consolidation Block HR2	HR2	AIEOP-BFM ALL	6
Consolidation Block HR3	HR3	AIEOP-BFM ALL	7
Consolidation Block HR4	HR4	AIEOP-BFM ALL	1
DAY33	d33	AIEOP-BFM ALL	2
other timepoints	other	AIEOP-BFM ALL/	22
		BFM-AML	
		and others	
**Timepoints VIE MRD**	**Abbreviation**	**Therapy Protocol**	**No. of Samples**
after 1st Induction	IND1/IND12	BFM-AML	19
after 2nd Induction	IND2	BFM-AML	2
after Consolidation 1	CON1	BFM-AML	6
after Consolidation 2	CON2	BFM-AML	6
post stem cell	pSCT	BFM-AML	4
transplantation			
other timepoints	other	BFM-AML	29

^1^ MRD negativity was proven using FCM-MRD as well as molecular methodologies (RQ-PCR, gDNA-PCR).

**Table A3:** Detailed clinical and biological characteristics of patients present in the data set used.

Patient	Type	Gender	Age	Fab Type	Genetics	Mutations
A	Relapse	w	7y 1m	M5a	KMT2A-	GATA2
					MLLT1	KRAS NRAS
B	Diagnosis	m	3y 3m	M7	CBFA2T3-	GATA2
					GLIS2	
C	Relapse	m	8y 10m	M7	DDX3X-	WT1 ETV6
					MLLT10	NRAS
D	Diagnosis	w	3y 9m	M1	NK	NPM1
						FLT3-ITD
E	Diagnosis	m	0y 1m	M5a	KMT2A-	nd
					MLLT3	
F	Diagnosis	m	17y 9m	M4	NK	FLT3-ITD
G	Diagnosis	m	1y 4m	M7	NUP98-	neg
					KDM5A	
H	Relapse	w	15y 3m	M5b	KMT2A-	neg
					MLLT3	
I	Diagnosis	w	17y 9m	sec. AML	KMT2A-	nd
					MLLT3	
J	Diagnosis	m	14y 11m	M7	KMT2A-	nd
					MLLT4	

**Table A4:** Details on the LAIPs identified in manual gating for the patients present in the data set used.

Patient	LAIP1	LAIP2	LAIP3
A	CD33/CD56 co-expression	↑ HLA-DR	
B	↓ HLA-DR	↓ CD38	
C	CD33/CD56 co-expression	↓ CD38	
D	↑ CD99	↓ HLA-DR	
E	expression of NG2+	↑ CD99	↑ HLA-DR
F	CD33/CD7 co-expression	↑ CD99	expression of CD123
G	↓ CD11a	↓ CD38	↓ CD371
H	↑ CD117	↓ HLA-DR	expression of CD123
I	↓ CD38	↓ CD371	expression of CD123
J	↓ CD371		

**Table A5:** Statistical summary of the amount of events and MRD percentage in the test data set used. CD34+/bermude and CD34+/bermude-predicted denote the statistics for CD34 positive events and events falling into the bermude area (CD34 neg) based on the ground truth and prediction by the Set-Transformer, respectively.

		Min	Max	Mean	Median
total	CFU	9502	440,160	204,544	190,463
	LAIP	12,516	408,700	173,526	161,543
MRD	CFU	0.0034%	45.61%	12.13%	2.96%
	LAIP	0.012%	70.25%	12.69%	2.58%
CD34+/bermude	CFU	2639	260,888	65,020	46763
	LAIP	6028	294,443	80,297	53,497
CD34+/bermude-predicted	CFU	5341	325,342	90,058	71,075
	LAIP	8597	321,073	89,711	63,474

### Appendix B.5. Experiments

In this section, a detailed description of the four experiments conducted as well as reasoning for the parameters chosen is given.

#### Appendix B.5.1. Set-Transformer

The same network architecture and hyper-parameters as in [17] were used, namely a sequence of three ISAB blocks with a row-wise linear layer on top, with i=16 induced points, a latent embedding dimension of d=32 and 4 attention heads for each layer. This is a supervised approach, hence a training and evaluation data set are required. Since samples with blast cells are scarce, a cross-validation was performed to maximize the amount of training and evaluation data. The model should be capable of detecting MRD in FCM data of new patients, meaning for a fair assessment samples taken from one patient must not be split up between the training, evaluation and test set. Therefore we conducted a “patient-cross-validation”, where samples taken from one patient form the test set and the rest of the patients are divided for training and evaluation so that the corresponding samples split up in approximately the fractions of 0.8 and 0.2, respectively. The architecture of the Set-Transformer can take a varying number of events per sample as input but requires a fixed length of the feature vector. As mentioned above, for many samples the backbone markers are not sufficient to successfully identify blasts and discriminate them from normal regenerative cells. On the other hand using all markers of the 8-color tube-specific panel of CFU (+drop ins) and LAIP tubes results in less training samples. Thus, there is a trade-off between the number of training data and the number of markers used. We test this method using only backbone markers as well as using tube-specific panel markers of CFU and LAIP tubes separately. A detailed listing of the number of evaluation, train and test samples is given in Table A6.

**Table A6:** Three experiments are performed separately, one with CFU data, one with LAIP data and one with backbone (BB) data. Available data sets are splitted into training, evaluation and test set. Each patient generates one split because the patients samples are hold out as test data (meaning that the rightmost column corresponds to the number of CFU, LAIP and backbone samples of each patient). All remaining samples from other patients are divided into training and evaluation set.

Patient	Marker	Train	Eval	Test
A	CFU	-	-	0
	LAIP	27	9	1
	BB	52	13	1
B	CFU	22	4	3
	LAIP	27	8	2
	BB	50	11	5
C	CFU	18	8	3
	LAIP	24	7	6
	BB	44	13	9
D	CFU	19	8	2
	LAIP	29	6	2
	BB	51	11	4
E	CFU	21	6	2
	LAIP	25	9	3
	BB	50	11	5
F	CFU	20	6	3
	LAIP	23	6	8
	BB	44	11	11
G	CFU	17	3	9
	LAIP	21	7	9
	BB	37	11	18
H	CFU	23	5	1
	LAIP	26	10	1
	BB	51	13	2
I	CFU	22	6	1
	LAIP	26	10	1
	BB	53	11	2
J	CFU	17	7	5
	LAIP	25	8	4
	BB	43	14	9

#### Appendix B.5.2. UMAP-HDBSCAN Classification Pipeline

The essence of the method proposed is to mix events of healthy cell populations (control-events) with the events to be classified (test-events) before UMAP is applied; it is crucial that the graph, on which the UMAP embedding is based, is constructed with control- and test-events pooled together. This way, differences of healthy cell populations between control- and test-events are smoothed out by the combination of the local distance metric with the push and pull characteristic of UMAP while optimizing the low dimensional embedding as explained in Appendix B.2. Healthy cell populations exhibit only small variations between patients; usually the location is stable and variations occur mainly in density as shown in Figure A1. Figure A2 shows the variations of the cell populations of the same patient at different time points of the therapy. The main differences are in the density of the cell populations, as different stages of regeneration occur at different times of therapy.

Our test set contains samples from different timepoints during and after therapy. To account for possible variations, 15 control samples are randomly chosen such that each timepoint is represented at least once (if available). Since for this method the number of control samples needed is quite low, the trade off between data and marker richness as discussed for the Set-Transformer above does not apply. Therefore, we use the 8-color tube-specific panel of CFU (+ drop ins) and LAIP tubes. The steps conducted to predict MRD in each test sample are described in detail in the next paragraphs.

First control-events are mixed with the events of the test sample. The control-events are sampled as follows:

1. Determine the total amount of events to be sampled from control samples.
  The number of control-events $n_{control\_total}$ mixed with the test sample varies with the number of its events $n_{test}$ and is determined by $n_{control\_total}=n_{test}\cdot control\_ratio$, where the ratio control_ratio is a parameter that was set to 0.8.
2. Select events from all available control samples.
  $n_{control\_total}$ is divided by the overall number of available control samples *M* to obtain the number of events to be selected from each control sample, i.e., $n_{control\_sample}=n_{control\_total}/M$. Using events from different control samples (in our case M=15) ensures a representative variety of phenotype expressions. Each control sample should have an equal contribution.
3. Every cell population should be represented in the control-events.
  Assuming we have cell population *A*, *B* and *C* with $n_{A}$, $n_{B}$ and $n_{C}$ cells, respectively, the proportion of events of each population sampled is $\frac{n_{control\_sample}}{n_{A}+n_{B}+n_{C}}$. The populations as defined in Section 2.2.1 are selected for sampling: monocytes, granulocytes, proerythrocytes, promyelocytes and CD34 positive cells. In addition, cells not belonging to either of these categories but falling in the bermude area are also selected.
4. Concatenate all selected events and shuffle them.

After the control-events have been sampled and pooled together with the test sample a 3D UMAP embedding is created. For this procedure default parameters settings are used except for the parameter min_dist, which is set to min_dist=0 allowing for denser packing of cells in the embedding space. This is recommended by the authors of UMAP, if subsequent clustering is performed. It was opted for a 3D embedding space as it is the maximum amount of dimensions that still provides the possibility of intuitive visualizations.

Once the events are embedded, clusters are detected with the HDBSCAN algorithm, where again all parameters were left at default settings except for min_cluster_size, which determines the minimum amount of events that can form one cluster. It was set to min_cluster_size=50, since samples with less than 50 blasts are denoted as MRD negative.

UMAP and HDBSCAN allows to represent and process unseen data without altering the learned mappings. This enables to transform new events into a previously learned embedding space and to assign these new events to the previously determined clusters. Due to UMAP properties, the embedding of unseen data into a fixed UMAP tends to loose significance, if these unseen data are not close to data from which the UMAP was generated. On the one hand, it is important that the UMAP is created with a reasonable proportion of test sample events. On the other hand, if there are to few control-events involved in the generation of the UMAP, these events will not lie close to the healthy populations of the test-sample and will produce unknown clusters falsely classified as blasts. Before predicting blasts based on the amount of control-events in each identified cluster, we therefore make use of the transform function and add additional control-events to the clusters in the embedding space. By doing so we maximize the ratio of control-events in non-blast clusters and hence lower the sensibility to the threshold used for classifying clusters as blast clusters. Those additional control-events are selected as described above, the number is again defined by a ratio, which we call transform_ratio to distinguish them from the first round of control-events sampled. We set transform_ratio=1, meaning that the same amount of events as present in the test sample are selected from the control samples and added to the learned embedding and clustering. If control-events dominate, blasts run the risk of adhering to non-blasts, which impedes identifying correct clusters as shown by Figure A3.

In general the ratios seem to not have a drastic effect as long as the test sample dominates the learned embedding and the variations of healthy cell populations are roughly covered by control-events. Therefore it was opted for a control_ratio a bit below 1 and a transform_ratio of 1 so that in total almost double as many control-events as test-events are randomly selected. Finally, all clusters identified by HDBSCAN that consist of less than 5% control-events are classified as blast clusters, meaning all events in this clusters are labelled as blasts and all others as non-blasts. In theory the blast clusters should exclusively contain events from the test sample, since control samples are blast free. However, this is usually not the case. If for example blasts are not entirely separated from healthy populations or due to events transformed into the embedding space at a later stage, some contamination of blast clusters with control-events can be observed. An empirical evaluation of hold-out samples suggested an impurity-acceptance-rate of 5% to be reasonable. By assigning a threshold rather than taking the cluster with the least control-events we open up the possibility of having no blasts as well as more than one blast cluster. More than one blast cluster can appear since blasts might differ in their CD34 expression or one biological meaningful blast cluster might be separated by HDBSCAN.

#### Appendix B.5.3. UMAP-HDBSCAN Classification Pipeline with Pre-filtering

The proposed approach relies on covering variations of non-blast populations by control samples. One way to reduce those variations beforehand is to distill test-events and discard events that can easily be predicted as non-blasts. As mentioned in Section 2.2.1 blasts can either be in the CD34 positive gate or the CD34 negative bermude gate. By eliminating events that are neither within these gates the search for blasts can be narrowed down. This is formulated as a supervised approach and realised with the Set-Transformer. Two models are trained, one to predict CD34 positive events and another to predict events in the CD34 negative bermude area. Since both types of cells are present in control samples no additional data are necessary and the models can be trained on those. Further, this classification task is not as demanding as predicting blasts and hence back bone markers are sufficient. To underpin this assumption an evaluation of the data-marker trade-off described above is given. The trained models are used to pre-filter the test sample where only events that are predicted by either one of the model are further examined. The remaining cells are classified as described in Appendix B.5.2. The pre-filtering adds another potential source of prediction error but reduces the run-time at inference as less events are embedded with UMAP.

#### Appendix B.5.4. UMAP-HDBSCAN Classification Pipeline-Variation of Control Samples

Even though the heterogeneity of healthy cell populations are not as severe as for blasts, there are some variations present as can be seen in Figure A1 and Figure A2. Initially only 15 control samples were chosen so that each stage of therapy is included roughly once. When increasing the number of control samples used, the number of events selected per sample decreases. The question is whether capturing more variety within one sample or between samples is of bigger importance. This is assessed by repeating the experiments of the proposed method above with double the amount N_control=30 and all control samples available.

### Appendix B.6. Alternative Clustering Methods

#### Appendix B.6.1. FlowSOM

Given the success of FlowSOM [51] and its common use within flow cytometry, we explore its clustering as an alternative to UMAP followed by HDBSCAN in our classification pipeline. We use the same hyper-parameters and meta-clustering as proposed in [51]. Since the goal is to predict rare cell populations we use a finer grid, namely a 20 × 20 grid instead of the default 10 × 10 grid, as suggested in [51]. While examining the method we identified the following issues. To detect blast clusters control-events need to be clustered together with healthy cells of the test sample. The combination of the local distance metric used by UMAP with the push and pull characteristic while optimizing the low dimensional embedding can smooth out differences of healthy cell populations between control- and test-events as explained in Appendix B.2. SOM, on the contrary, uses a global distance metric during training and for assigning a *best matching unit* (BMU) of the grid to each event in the high dimensional feature space. Hence, control-events, albeit mostly next to the corresponding BMU of healthy cells of the test sample, tend to get assigned to separate BMUs. In the meta-clustering step of FlowSOM, where node centers are clustered, those neighboring BMUs can be clustered together counteracting this issue. However, we noticed that nodes representing blasts are likely to be consumed by neighboring nodes resulting either in not being detected or increasing the false positives. Another solution is to use a smaller grid, where healthy cells of control and test samples are forced to get assigned to the same node, but then the issue of impure blast nodes arises since MRD can be as low as 0.0034% in test samples (see Table A5). Further, we noticed that the impurity of nodes corresponding to blasts leads to unsatisfactory results even if the node is correctly detected as blast cluster. While FlowSOM has proven to be a successful tool for FCM data visualization and exploratory data analysis that can aid manual gating, self-organizing maps as a stand alone clustering and in combination with meta-clustering are not well suited for our specific classification pipeline.

#### Appendix B.6.2. PhenoGraph

The runtime of PhenoGraph [52] was significantly higher than for UMAP + HDBSCAN: to create a clustering for 400,000 events it took almost 40 min; in our classification pipline we need to process more than 1Mio events (including control-events) if a sample is constitued of 400,000 events, PhenoGraph was thus considered as impractical.

#### Appendix B.6.3. Stochastic Neighborhood Embedding (SNE)

UMAP offers some advantages over SNE methods (for example t-SNE [47]) such as superior run-time and more intuitive hyper-parameters, with equal or better results. See [15] for a comparison between UMAP and t-SNE. We chose UMAP as a representative of recent manifold learning methods.

### Appendix B.7. Technical Details

All our code is setup in python v3.8.10. For the Set-Transformer approach we used pytorch v1.10.0 + cu102, for the classification pipeline we used the python packages umap-learn v0.5.1, and hdbscan v0.8.27. For FlowSOM we adapted the implementation of the python package cytopy v2.0.1 to our needs. Plots were created with the python package matplotlib v3.4.1.

The classification pipeline experiments were all conducted on an in-house server with 28 cores and 128 GB RAM. The Set-Transformer models were trained on an GeForce GTX TITAN X with 12 GB RAM.

Training for each Set-Transformer model in the context of patient cross validation (Section 3.1) took between 20 and 30 min, predicting blasts approximately 10 seconds. For the pre-filtering of test samples (Section 3.3) two Set-Transformer models were trained; one for predicting events in the CD34 negative bermude area and one for predicting CD34 positive events—the training duration was 5 h 41 m 43 s and 3 h 52 min 36 s, respectively. Predicting the corresponding events took approximately 10 seconds. Training the models is a one time necessity, at inference only the time needed for prediction is relevant. The classification pipeline proposed omits this classical training phase; Table A7 shows the average run-time needed to predict blasts for separate parts of the pipeline. Pre-filitering samples does not only improve performance but also speeds up the classification process significantly.

**Table A7:** This table gives an overview of the average run-time per sample for the full sample and for the pre-filtered sample.

	Avg	Avg Pre-Filtered
UMAP-fit	6 m 40 s	2 m 53 s
UMAP-transform	7 m 59 s	3 m 33 s
HDBSCAN-fit	25 s	9 s
HDBSCAN-transform	51 s	18 s
total	15 m 55 s	6 m 53 s (+10 s pre-filtering)
